# Addressing the socioeconomic divide in computational modeling for infectious diseases

**DOI:** 10.1038/s41467-022-30688-8

**Published:** 2022-05-24

**Authors:** Michele Tizzoni, Elaine O. Nsoesie, Laetitia Gauvin, Márton Karsai, Nicola Perra, Shweta Bansal

**Affiliations:** 1 ISI Foundation, Turin, Italy; 2 Department of Global Health, School of Public Health, Boston University, Boston, MA USA; 3 Center for Antiracist Research, Boston University, Boston, MA USA; 4 Department of Network and Data Science, Central European University, 1100 Vienna, Austria; 5 Alfréd Rényi Institute of Mathematics, 1053 Budapest, Hungary; 6 School of Mathematical Sciences, Queen Mary University of London, London, UK; 7 Department of Biology, Georgetown University, Washington, DC USA

**Keywords:** Infectious diseases, Computational models

## Abstract

The COVID-19 pandemic has highlighted how structural social inequities fundamentally shape disease dynamics. Here, the authors provide a set of practical and methodological recommendations to address socioeconomic vulnerabilities in epidemic models.

## Teaser

The COVID-19 pandemic has highlighted how structural social inequities fundamentally shape disease dynamics, yet these concepts are often at the margins of the computational modeling community. Building on recent research studies in the area of digital and computational epidemiology, we provide a set of practical and methodological recommendations to address socioeconomic vulnerabilities in epidemic models.

## Socioeconomic factors in infectious disease modeling and surveillance: the need for a comprehensive approach

The investigation of social determinants of health and disease stands at the core of social epidemiology, a discipline whose tradition dates back to the mid-20th century^1^. Social conditions and in particular social disparities related to income, wealth, race, ethnicity, gender, and education, to cite only a few, are known to affect the health status of individuals and they reflect in unequal health outcomes when it comes to disease burden^2^.

In recent decades, significant effort has been devoted to investigating the relationship between differences in socioeconomic conditions and the prevalence of non-communicable diseases^3^; however, the socioeconomic divide represents a key factor in the spread of infectious diseases as well. The effect of socioeconomic status (SES) on the spread of respiratory infections has been recognized in several past and recent epidemics, for instance in the case of the 1918 and 2009 flu pandemics lower SES was found to be associated with the highest disease burden^4–6^. Similarly, socioeconomic disparities, such as unequal access to care and sanitation, have been shown to be important in the West African Ebola outbreak^7^ and in the spread of vector-borne diseases, such as malaria^8^ and dengue^9,10^.

The COVID-19 pandemic has revealed and further exacerbated such differences across several dimensions. During the pandemic, health outcomes have been significantly different by social strata, with inequalities in the distribution of infections, hospitalizations, and deaths closely matching income, occupational and racial disparities^11–13^. In the early phase of the pandemic, such inequalities were strongly linked to the affordability of non-pharmaceutical interventions (NPIs), as the feasibility of adopting prolonged social distancing measures has been a privilege of a few^14,15^. Following the rapid development of efficacious vaccines, inequities in health outcomes have been further driven by disparities in vaccine distribution, and accessibility, especially across the Global North-South Divide^16^.

While particular disparities in health outcomes were not obvious at the start of the pandemic, and many others are yet to be discovered, the populations that have borne the greatest morbidity and mortality burden are the same populations that tend to have the highest burden of disease and limited access to optimal healthcare. These disparities are largely driven by structural factors and therefore require collaboration with social scientists, historians, and economists to understand the impact of past and current factors on the health outcomes of these populations, and design studies that focus on addressing underlying factors rather than unjustly blaming the individuals affected.

Despite the clear understanding of the importance of socioeconomic inequalities in disease transmission dynamics, the epidemic modeling community has often neglected these aspects in traditional mathematical approaches. One reason is the lack of an empirically driven mechanistic description of the interaction between inequalities and disease outcomes. Most mathematical models account for variations in health risk by age, and by occupational status, often limited to the dichotomy of student/worker. Contact heterogeneity, which is known to be key in defining the risk of infection, is usually assumed to be encoded into the demographic structure of a population^17^; therefore, implying that countries or regions with similar demographics will experience similar epidemic trajectories. Despite their extensive and successful application in many real-world settings to infer key epidemiological parameters, evaluate different epidemic scenarios and inform public health interventions, many computational models remain agnostic about socioeconomic disparities, and they provide, by definition, only partial views of transmission mechanisms at play. However, as models are becoming more and more a standard tool for decision makers to inform public health policies, such as the adoption of NPIs for entire populations, this might in turn lead to widening social and health inequities. For example, models that assume that the risk of exposure and infection is the same for every individual in the population could lead to the implementation of interventions that are the same across a population irrespective of the social factors that create unequal exposure. Depending on the assumptions, such interventions will likely favor one group more than another.

In recent years, researchers have advocated for the extended use of computational and digital tools to tackle the emergence of novel infectious diseases and to rapidly face new outbreaks^18,19^. More recently, the importance of including social aspects in infectious disease modeling has been highlighted by numerous authors^20–22^. In this comment, we argue that the field of digital and computational epidemiology may remedy some of the challenges of socioeconomic inequalities in outbreak science. We provide some relevant examples of studies that demonstrate the opportunities of digital approaches to these issues and we conclude by making a set of practical recommendations to advance the field toward a more comprehensive approach.

## Computational and digital epidemiology approaches to address the socioeconomic divide in the COVID-19 pandemic

Despite the challenges posed by socioeconomic inequalities to disease modeling and surveillance, the COVID-19 pandemic has also shown the promise of computational and digital epidemiology to address these gaps. Indeed, key insights into the effects of social inequalities during the COVID-19 crisis have come from the analysis of novel digital traces and their integration into epidemic models. Through the analysis of mobility patterns derived from de-identified mobile phone data, several studies have revealed that individuals belonging to higher SES could better afford the adoption of health-protecting behaviors, such as reducing their mobility and social distancing^15,23,24^. As a consequence, disadvantaged groups that were not able to limit their social interactions experienced the highest rates of infections. Socioeconomic constraints to mobility reductions were associated with income levels, especially in the USA^15,25^, but also more broadly with the structure of the labor market, as found in France^26^, Italy^27^, and Colombia^28^. In general, workers in informal sectors, or in essential services, such as agricultural workers, were incentivized or required to continue working away from home despite the restrictions, leading to higher infection risks.

By exposing the hard social constraints that limited the adherence to physical distancing in many countries, researchers have underscored the need for more equitable policies in response to COVID-19 and provided practical guidance to achieve them with the aid of computational models. For instance, by mapping the movements of about 100 million people to half-million points of interest in the US, and creating a network model of SARS-CoV-2 based on these data, Chang and collaborators identified a range of optimal reopening strategies to minimize the burden of infections among the most deprived populations^29^. A modeling study of the spread of SARS-CoV-2 in Santiago de Chile highlighted how the deep socioeconomic inequalities of the Santiago population, and the associated disparities in mobility reductions, significantly delayed the end of the first COVID-19 wave^30^. Counterfactual scenario simulations showed that a more equitable social distancing would have prevented more than 80% of deaths reported in Santiago, in the same period. Similarly, by combining detailed mobility data describing contact rates between households in the metropolitan area of Philadelphia, with a computational model of epidemic spread, Nande et al. demonstrated that evictions, which would inevitably follow mass unemployment due to the COVID-19 closures, lead to a significant increase in COVID-19 cases^31^. Such an increase would mainly affect denser and poorer neighborhoods of the city, widening the disparities in health risks associated with the pandemic. As a consequence, eviction moratoria could be an effective public health measure to avoid rapid surges in COVID-19 cases.

All these modeling efforts usually incorporated socioeconomic factors effectively, through the integration of behavioral data, such as mobility traces, to calibrate classic age- and spatially structured epidemic models. Fewer studies, instead, defined an epidemic model where socioeconomic disparities are encoded into its mathematical formulation. For instance, Ma et al.^32^ developed a compartmental model with assortative mixing derived from the census distribution of ethnic groups in the city of New York to explain the high burden of disease in minority populations.

While both approaches either indirectly (the former) or directly (the latter) take into account socioeconomic disparities, only the latter offers the opportunity to actively investigate the mechanisms of infection inequality, and identify strategies to prevent them.

**Fig. 1:**
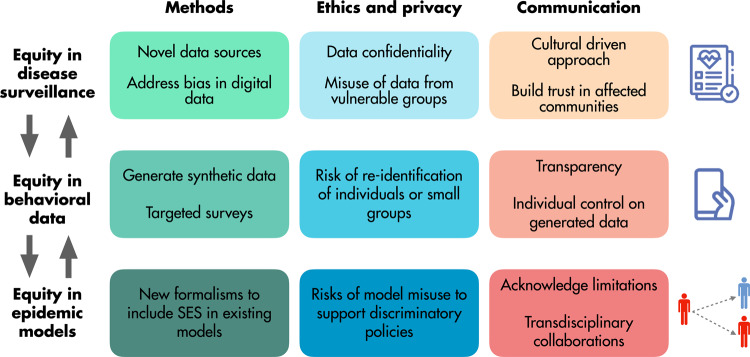
Practical guidelines to introduce an equity lens into the computational modeling of infectious diseases and related challenges. The figure illustrates and summarizes key recommendations and associated challenges, grouped by area of relevance in the data-model framework of computational epidemiology: surveillance data, behavioral data, and epidemic models. SES socioeconomic status.

Building on these examples, in what follows, we make a set of specific recommendations to advance the field by bringing the concept of socioeconomic equity to all the three main aspects that stand at the core of the data-model loop in computational epidemiology: surveillance data, behavioral data, and epidemic models (see Fig. 1).

### Equity in disease surveillance

Disparities in exposure, susceptibility, transmission, and treatment lead to certain populations bearing a higher disease burden than others. These disparities are driven by unequal access to resources that promote health at the individual and community level (i.e., social determinants of health), discrimination against lower SES, displaced populations (e.g., refugees), and structural racism against people of color^33–35^. To address these disparities, we need reliable data on infection burden and access to public health resources for different socio-economic groups. Such data are critical to the prevention of infection and to characterize the disparate impacts of interventions across social and economic strata that can create or exacerbate health inequities. We, therefore, need to design disease surveillance systems that are focused on promoting equity.

Incorporating an equity lens into surveillance systems would differ depending on the data sources, and local context. Digital data collected from high-volume healthcare data, participatory surveillance systems, or from mining digital traces from social media, mobile phone usage, and Internet usage are opportunistic in nature meaning they are not usually generated for disease surveillance purposes. Such novel data streams can improve timeliness, spatial and temporal resolution, and provide access to unreachable populations for effective infectious disease surveillance, but such data must be used with caution and with explicit equity lens^36–39^. Digital healthcare records for passive syndromic surveillance, for example, are dependent on healthcare accessibility, healthcare-seeking behavior, and other reporting issues^40^. Studies in the US for influenza-like illness have demonstrated that disease burden can be underestimated in low SES populations from healthcare-based surveillance^41,42^, which not only produces misleading estimates of disease burden but also underestimates the extent of health disparities. Until healthcare systems reach equity, statistical approaches must be developed to account for the measurement biases and quantify uncertainty in such data for infectious disease modeling applications^42^.

Demographers and social scientists have been developing methods to account for bias due to nonrepresentative samples. For instance, in the early work by Zagheni and Weber^43^, the authors accounted for variations in Internet penetration rates to correct their migration estimates based on e-mail data. Similar approaches could be extended to the field of digital epidemiology. In some cases, it is not always possible to address the bias inherent in digital data. Therefore, communicating these biases in a way that is easy for the audience (i.e., the public and policymakers) to understand is important.

Addressing limitations in disease surveillance systems requires a community and cultural context-driven approach to data collection. For example, knowing that Black or African American people have a higher prevalence of the risk factors associated with severe COVID-19 disease should have prompted action and policy to collect consistent racial data at the beginning of the pandemic in the United States to identify and effectively respond to these disparities. Unfortunately, that was not the case. As of September 2020, only New York State was reporting race data for 100% of COVID-19 cases, while this percentage was zero for many other states^44^. These disparities are often driven by discrimination and bias that is well documented but might not always be discussed in the context of disease surveillance. Collaborating with public health scientists who study health disparities, historians and social scientists with expertise in the factors that have long influenced health outcomes, and community leaders is extremely important. However, the context and individuals represented will differ by country and region.

Furthermore, racial and ethnic discrimination has impacted the trust of healthcare providers within certain communities. It is therefore our responsibility as public health officers and researchers to earn the trust of these communities to enable the submission and collection of data for public health surveillance. A first step to developing equitable disease surveillance systems is designing policies that focus on addressing health disparities. A focus on health disparities will then require data that captures populations affected, disaggregated by social identity groups and social status. While aggregated data can demonstrate associations between disease burden and SES, disaggregated data is critical to accurately measuring health disparities. Second, data collection should also focus on capturing the interaction between different social processes that interact to create disadvantages. Individuals from low SES might have other social disadvantages depending on the context. In the US, for example, SES tends to intersect with certain racial or ethnic identities. While in other countries, low SES might be higher for vulnerable populations such as refugees. We acknowledge that SES interacts with different infectious diseases in varied ways; therefore, it is important to determine what SES factors are relevant for a particular infectious disease. For example, improving some SES factors might lead to improved access to quality housing, which might reduce the incidence of some respiratory conditions. However, improved housing is not associated with the reduced spread of all infectious diseases. Third, modelers and analysts should conduct studies to inform the detail and structure required in disease surveillance data for effective computational disease modeling studies focused on health disparities. And importantly, fourth, policies must be created to enforce protection and deter misuse of data from minority, underserved and under-resourced populations.

### Equity in behavioral data

The COVID-19 pandemic has shown that human behavioral patterns are an essential factor to consider for the better understanding, modeling, and forecasting of an ongoing epidemic. As equity in disease surveillance data is needed to identify disparities in disease exposure and transmission, equity in behavioral data is needed to address such disparities in statistical and computational epidemic models. We identify three main directions for action to integrate equity into behavioral data that are relevant to infectious disease dynamics.

First, socioeconomic differences in behavioral patterns could be captured by leveraging existing socioeconomic data sources, such as routinely collected census survey data.

Socioeconomic data underlying the observed disparities in health outcomes, such as income distributions, or population stratifications by racial and ethnic groups, are generally available in several countries, from official statistical sources, at very high spatial resolutions, and simple interaction models can be developed with them. As an example, the IPUMS International Historical Geographic Information System^45^ represents a rich source to describe the distribution of essential or frontline workers—who are significantly more exposed than other groups^46^—across subpopulations. Similarly, the American Community Survey provides population numbers by race or ethnicity, at the census block level, that can be used to define varying rates of exposure or mixing patterns for different identity groups^32^. Despite their potential biases, these data sources provide usually the most accurate socioeconomic indicators available in several countries.

A second approach could be combining novel digital data sources and census data to generate synthetic data. Digital trace data can be collected in an aggregate way to preserve individual privacy and further calibrated to match the socioeconomic structure reported by census, as done by Replica to generate racial disaggregated mobility patterns^47^. The pandemic emergency has encouraged large tech companies to release digital trace data, in particular near real-time movement data: mobility reports provided by Google and Apple, mobility maps shared by Meta through its Data for Good Program, and mobility indicators made available by location intelligence companies, are all valuable inputs for epidemic models^48^. Future work should focus on developing the most statistically appropriate methods to combine such data streams to capture a refined picture of subpopulations. Efforts in this direction have started by building remotely sensed socioeconomic maps in several countries combining multiple data sources like mobility, satellite, night-light emission, or online social media, but still much research is needed^49–52^. Both traditional statistical approaches such as Iterative Proportional Fitting, Monte Carlo sampling, and machine learning techniques such as Self-Organized Maps or Generative Adversarial networks are appropriate candidates to generate synthetic behavioral data from novel data sources^53–55^. The synthetic and inferred data could then be used to represent mobility or contact patterns of subpopulations that will feed epidemic models, in a similar way to what is customarily done for age-dependent contact matrices^56,57^.

Finally, targeted data collections should be devised to supplement behavioral data that are passively collected. Although digital data represent a powerful tool to measure behavioral changes during an epidemic, they commonly suffer from observational biases. They can provide insights only about people who have access to digital services, and thus overlook deprived socioeconomic groups. In some cases, active data collection might be the only effective approach to faithfully capture behaviors of underrepresented communities, as called for by the United Nations’ Sustainable Development Goals (SDG 17.18). As an example, during the COVID-19 pandemic, UNICEF and the Harvard Humanitarian Initiative developed the Community Rapid Assessment to map protective behaviors across different groups in rural and urban areas through mobile phone surveys^58^. Traditional contact surveys should collect SES information, and new survey campaigns should be focused on low-resource settings^59^. Also, the deployment of proximity sensors^60^ or GPS trackers^61^ in urban and rural communities represents an alternative to measure contact patterns relevant to disease transmission in hard-to-reach, low-income settings.

### Equity in epidemic models

Modeling frameworks that include SES at their core are largely missing and urgently needed^22^. When thinking about possible solutions, it is important to realize how one-fits-all approaches are hardly conceivable. In fact, the details of the implementation are likely to be a function of the data available as well as the type and scale of the model considered.

Arguably, the simplest concrete step in this direction would be extending standard compartmental structures to accommodate different SES as input. Similar to what is customarily done for age, compartments could be stratified by SES. Hence, models for the same disease, but developed for different countries, will reflect not only different age-pyramids but also different socioeconomic structures. Such an approach would allow accounting for differences in healthcare access and for heterogeneities in ability to respond to NPIs across SES. The next step could be capturing the stratification of contacts across both age and SES. Hence, we would move from classic contact matrices *M*_*k,j*_ (describing the contact rate of individuals in age bracket *k* with those in age *j*) to ${M}_{{k}_{\alpha }{j}_{\beta }}$ (describing the interactions of individuals of age *k* and SES *α* with individuals of age *j* and SES *β*). Such models would allow capturing the correlations in contact patterns and their variations induced by NPIs as a function of socioeconomic indicators.

Some epidemic models are spatially aware and include the coupling between subpopulations (i.e., neighborhoods, regions, countries) through human mobility patterns. Such models could be extended to account for the interplay between SES and movements. To this end, the mobility matrices, describing the travel rates across subpopulations, could be stratified by SES, including the number of individuals of a given SES, traveling between location *i* and location *j*. Such extension would provide a more realistic description of human mobility and its variation induced by NPIs. Furthermore, it would allow us to estimate the impact of mitigation measures that target mobility reduction, considering different abilities to comply across SES.

As well-mixed compartmental models often neglect relevant population heterogeneities, other types of models may be needed to describe disease spread with sufficient detail. Agent-based models are the most detailed and complex modeling frameworks. They are based on generating synthetic populations that account for households, workplaces, and schools. Socioeconomic indicators could be used as additional households’ features, together with their composition in terms of age and size. These models could be adopted to study the effects of school closures, household mixing, and remote working across different SES and to design interventions that account for inequalities to a higher level of realism.

Each of the proposed directions is of course far from trivial and underlies a clear increase in the complexity of models, some of which are already scratching the boundaries of what is computationally feasible. Furthermore, such extensions will give rise to and describe a wide range of mechanisms, dynamics, and interactions that do not have yet a solid theoretical basis. Just to offer an example, adherence to NPIs is a complex phenomenon that has been linked to age, gender, education, political beliefs, country of residence, and SES. Hence, in absence of precise data, more expressive models like those we are advocating for would need extra layers of assumptions and parameters.

Furthermore, SES is an aggregated indicator encompassing a wide range of factors that can, directly or indirectly, affect disease spreading and outcomes. Overcrowding in households and workplaces, limited access to healthcare and vaccinations, and limited ability to comply with NPIs by reducing contacts and mobility patterns due to job security or type are just a few examples. Hence, another concrete step toward including inequity in epidemic models is to focus directly on such factors and study the differences they induce across SES as an emergent phenomenon rather than input. Differences in the number of contacts and interactions across groups due to overcrowding could be investigated via compartmental models that allow for differences in the effective transmissibility of a pathogen. The effects of job security and type could be investigated in spatially aware models linking them to variations in mobility patterns. The impact of overcrowding in workplaces could be investigated via agent-based models. Such an approach targets specific causes and mechanisms associated with SES, that might lead to inequalities in disease spreading. It would allow developing a better understanding of their impact on diseases on one side while offering a natural testbed to design specific interventions on the other. As such, it is complementary with respect to what we have described above where SES is considered as an input for the models.

Epidemic models that account for SES as input or that explicitly consider specific drivers of disease transmission associated with SES in their formulation would also allow us to formulate and address new questions. For example, they would enable us to study the impact of health disparities under different (controlled) conditions, disentangle the effects of multiple, and competing, drivers of transmission on health disparities, and design intervention strategies that consider the overall burden as well as health inequality^42,62–67^.

## Ethics and privacy challenges

We acknowledge that pursuing the above recommendations implies facing some relevant ethical challenges, which should be carefully considered by infectious disease modelers and researchers from multidisciplinary teams before they embark upon research on the topic.

Design of disease surveillance efforts should always aim at protecting the confidentiality of personal information under specific legal safeguards against the risks of disclosure. Individuals should be allowed to opt-out of public health surveillance activities if deemed at risk. The use and share of non-aggregated surveillance data should require the approval of trained research ethics committees^68^. Public health data collection must be conducted in a transparent and accountable manner, minimizing the risk that subjects of public health surveillance may face discrimination.

Similarly, the collection, analysis, and sharing of behavioral indicators from digital traces, such as mobile phone data, should adhere to the highest standards of anonymization, preventing data misuse that could potentially lead to re-identification of individuals or small groups. Differential privacy schemes, based for example on the addition of noise to the original data, should be applied to all data releases^69^. Furthermore, empowering individuals by providing them with more control over the data they generate, for instance through the creation of a Personal Data Store, could represent a solution to the inclusion of marginalized communities in behavioral data collection efforts^70^.

Finally, epidemic models of infectious disease spread should be transparent in their assumptions, and the interpretation of their results should always minimize the risk of stigmatization of vulnerable communities. Modeling efforts should never be used to support the adoption or enforcement of discriminatory policies.

In conclusion, we acknowledge that, in some circumstances, benefits may not always outweigh the risks and all modeling studies that focus on minorities or marginalized communities should be preceded by systematic risks and harms assessment, following best practices such as the guidelines provided by UN Global Pulse^71^.

## Conclusion

The COVID-19 pandemic has demonstrated that socioeconomic inequalities cannot be ignored when it comes to understanding the distribution of disease burden, the behavioral responses to the epidemic, and the entire epidemic dynamics. An equity-focused approach to computational modeling of infectious diseases requires critically assessing how the data, modeling assumptions, and recommended policies impact individuals from different SES. In addition, communicating findings from modeling studies during public health emergencies remains a challenge. Especially, how to quantify and communicate uncertainty to policymakers and the general public. Addressing this challenge requires collaboration with communication experts and community leaders to develop culturally appropriate messages. In addition, the humility to acknowledge limitations in modeling and the compassion to understand the social and political processes that drive individual decision making can go a long way in impacting responsiveness to information communicated during public health emergencies.

In this comment, we have made recommendations on how we can develop approaches to improve the collection and use of surveillance and behavioral data, and how we could incorporate socioeconomic information into epidemic models. While not comprehensive, we hope these recommendations will lead to constructive conversations around the need for digital and computational approaches that are inclusive and focused on reducing rather than exacerbating health disparities during public health emergencies.
